# RELATED FACTORS ON HOSPITALIZATION OF NURSING HOME RESIDENTS IN KOREA USING QUASI-POISSON REGRESSION

**DOI:** 10.1093/geroni/igad104.2298

**Published:** 2023-12-21

**Authors:** Juh Shin, Sun Ok Jung, Eun Jeong Min, Jung Eun Kim

**Affiliations:** George Washington University, Washington, District of Columbia, United States; Ewha Womans University, Seoul, Republic of Korea; The Catholic University of Korea, Seoul, Republic of Korea; California State University, Los Angeles, Los Angeles, California, United States

## Abstract

The appropriateness of hospitalization for nursing home (NH) residents is still debated, with factors such as timeliness, available treatment, healthcare staff, medication options in hospitals, and safety issues determining appropriateness. Although factors impacting hospitalization have been studied expansively, research has been limited to nurse staffing. This study aims to investigate how organizational factors, nurse staffing, and government incentives impact hospitalization due to infection among NH residents in Korea. A cross-sectional design was used, and data were collected via survey from a total of 51 NHs from August 27, 2021 to March 25, 2022 using quota sampling. A total of 35 explanatory variables were included, and the response variable was the count of hospitalized residents due to infection. We analyzed data using quasi-Poisson regression. The average number of residents of hospital transfers due to infection was 5.22 (SD 7.99) per nursing home. The number of residents with Alzheimer’s or dementia increased by 1 person, and the number of hospitalized residents due to infection increased by 1.01. Moreover, we found that in NHs that did not receive top-grade incentives due to good performance, the number of residents admitted to the hospital due to infection decreased by Exp (b) 0.5133. More stable, specialized advanced nursing staff and leaders specializing in high-quality care for residents with Alzheimer’s or dementia were more effective in decreasing hospitalization. The measure of hospitalization in the future should focus on quality improvement, as hospitalization is universal use for many countries by extricating necessary versus avoidable hospitalization.

